# A Twist in the Tale: A Case Report on the Laparoscopic Management of a Rare Gallbladder Torsion

**DOI:** 10.7759/cureus.97543

**Published:** 2025-11-23

**Authors:** Sunday O Oyeniyi, Kumarakrishnan Samraj, Opeyemi Omonijo, Tobiloba Ajibola

**Affiliations:** 1 Surgery, Torbay Hospital, Torquay, GBR; 2 General Surgery, Wexham Park Hospital, Slough, GBR; 3 Family Medicine, Federal Medical Center Abeokuta, Abeokuta, NGA; 4 Family Medicine, Lagos State Primary Healthcare Board, Lagos, NGA

**Keywords:** acute cholecystitis, gallbladder torsion, laparoscopic cholecystectomy, low bmi, rare surgical condition

## Abstract

Gallbladder torsion is a rare but potentially life-threatening surgical emergency that poses a significant diagnostic challenge due to the condition's non-specific clinical presentation and often inconclusive imaging findings. We present the case of a 53-year-old woman with sudden-onset right upper quadrant abdominal pain and unintentional weight loss. Her low body mass index and initial clinical findings suggested acute cholecystitis. Ultrasonography revealed a distended gallbladder without cholelithiasis. Despite conservative management, her symptoms worsened, prompting an emergency diagnostic laparoscopy. Intraoperatively, a 360° clockwise torsion of a completely mobile, ischemic gallbladder was identified. Detorsion followed by laparoscopic cholecystectomy was performed, resulting in an uneventful recovery for the short-term follow-up period of three months. Gallbladder torsion should be considered in middle-aged or elderly patients presenting with atypical features of acute cholecystitis, particularly when imaging is inconclusive and there is a poor response to medical therapy. Early recognition and prompt surgical intervention are essential to prevent serious complications. This case highlights the importance of maintaining a high index of suspicion for rare causes of acute abdomen.

## Introduction

Gallbladder torsion is a rare but potentially life-threatening condition where the gallbladder twists on its mesentery along the axis of the cystic duct and artery, leading to compromised vascular supply, ischemia, necrosis, and potentially perforation. Since its first description by Wendell in 1898 [1], fewer than 600 cases have been documented in the literature, based on pooled case reports, with a striking predominance in elderly women [2-4]. The condition poses a diagnostic challenge due to its non-specific clinical presentation that closely mimics acute cholecystitis. The rarity of this condition, combined with its clinical similarity to acute calculous cholecystitis, makes preoperative diagnosis difficult. Radiologic features may suggest torsion, but a definitive diagnosis is often only made intraoperatively [2,5]. Preoperative recognition is difficult, and definitive diagnosis frequently occurs intraoperatively. Understanding these anomalies is crucial for surgical planning, as failure to identify them may lead to complications such as bile duct injuries or delayed treatment. Together, these reports emphasize the importance of careful preoperative imaging and a high index of suspicion for atypical gallbladder pathologies to ensure safe and effective patient outcomes [6].

Anatomically, gallbladder torsion occurs when the gallbladder rotates along the axis of the cystic duct and artery, usually due to an abnormally long or underdeveloped mesentery that allows the gallbladder to become “free-floating.” This can lead to ischemia, necrosis, and ultimately perforation if not promptly addressed. Two types of anatomical variants have been described: type I (incomplete torsion), in which there is a partial attachment to the liver, and type II (complete torsion), where the gallbladder is entirely suspended by the cystic duct and artery [4]. In terms of the degree of rotation, torsion can also be classified as complete (greater than 180 degrees, as in our case) or incomplete (less than 180 degrees) [4,7,8]. The direction of rotation may be influenced by adjacent organ peristalsis; clockwise torsion possibly triggered by colonic peristalsis, and anticlockwise torsion by gastric peristalsis [9]. Delay in diagnosis and treatment of gallbladder torsion can result in gallbladder necrosis, perforation, biliary peritonitis, and even death [7].

Here, we present a case of gallbladder torsion in an elderly woman who was successfully managed laparoscopically. This case highlights the diagnostic dilemma and the importance of early surgical intervention in suspected atypical gallbladder pathology.

## Case presentation

**Figure 1:**
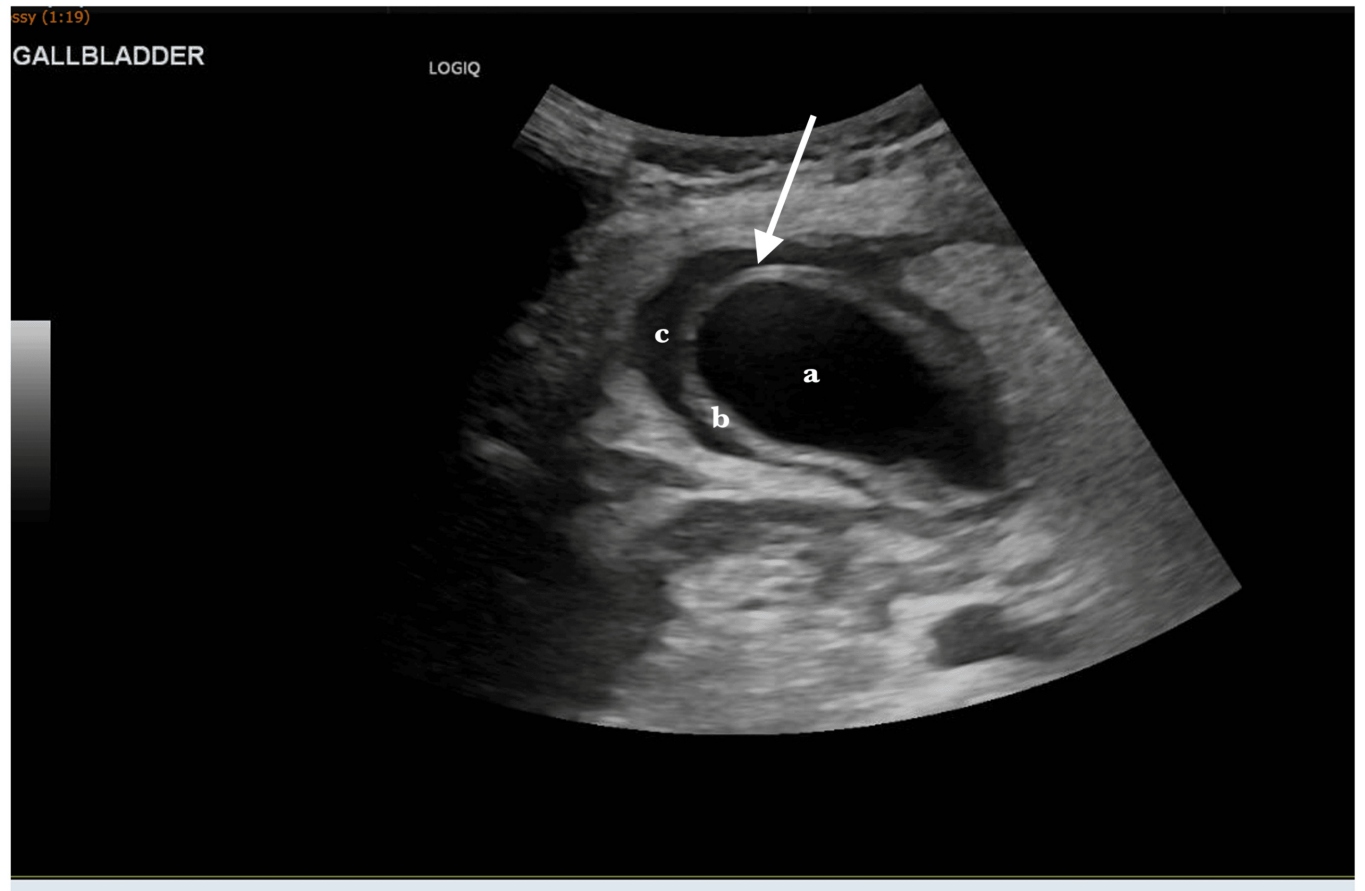
Abdominal ultrasound revealing gallbladder structures (arrow). Transabdominal ultrasound image demonstrating a markedly distended gallbladder (a) with diffuse wall thickening (b) and a hypoechoic rim of pericholecystic fluid (c), consistent with inflammatory changes. No echogenic foci or posterior acoustic shadowing were identified to suggest gallstones.

**Figure 2:**
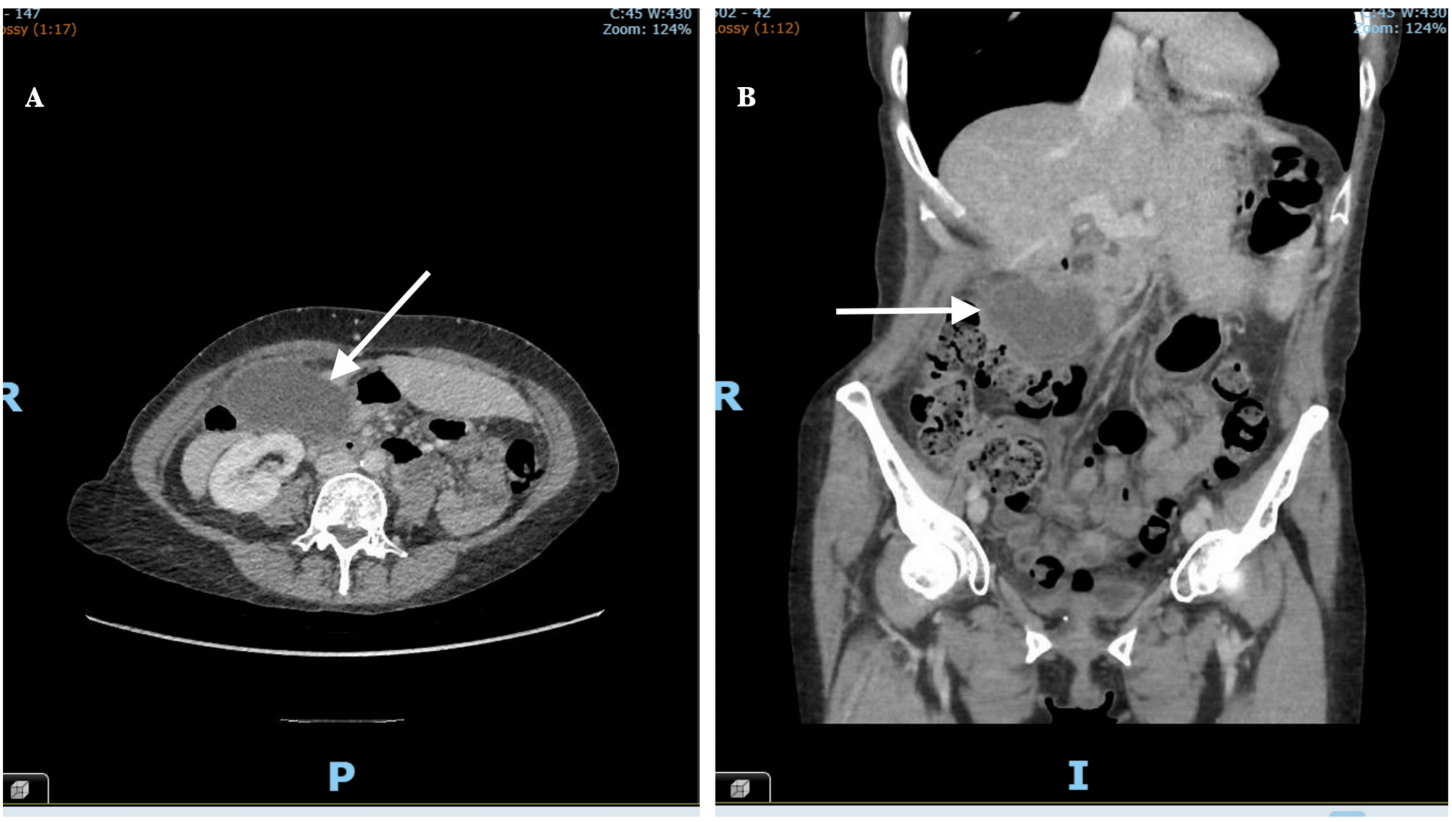
Computed tomography scan showing a thickened gallbladder wall, with no perforation or dilation (A) and (B) Contrast-enhanced computed tomography (CT) images in axial (A) and coronal (B) planes demonstrating a thickened gallbladder wall (arrows) without evidence of perforation or biliary dilation. The surrounding fat planes are preserved, and no gallstones are identified.

A 53-year-old woman presented with a history of sudden onset right upper quadrant pain of about 18 hours duration prior to presentation. The pain was described as sharp, constant, and radiated to the right shoulder and was associated with nausea and vomiting. The patient had a positive history of weight loss (lost about 6.35kg in the last month), per vaginal bleeding and BMI noted to be low (17.46 kg/m^2^). She denied having a fever or altered bowel habits. There was no past medical history or diagnosis of gallstone pathology or abdominal trauma or previous abdominal surgery. On examination, she had no obvious abnormality, non-toxic presentation, no abdominal mass felt and no pulse-temperature dissociation. She was hemodynamically stable but had mild leukocytosis (13,800/mm³) and mildly elevated liver enzymes. Abdominal ultrasound showed a distended gallbladder with wall thickening and pericholecystic fluid but no gallstones (Figure 1). A CT scan performed during this initial presentation confirmed a thickened gallbladder wall without perforation or biliary dilation (Figure 2). She was managed conservatively and discharged following clinical improvement.

**Figure 3:**
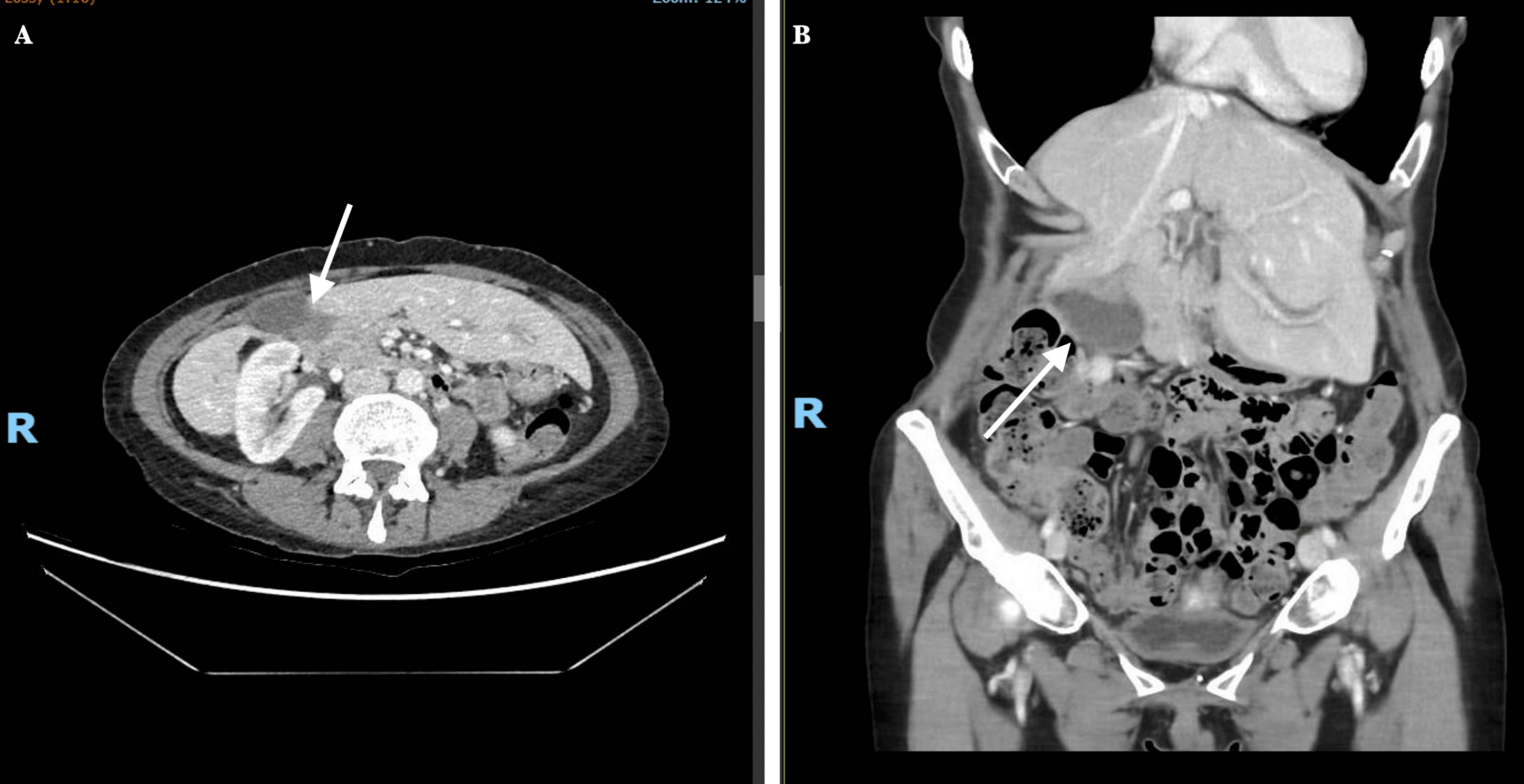
Repeat CT scan of gallbladder during the second presentation Repeat scan contrast-enhanced computed tomography (CT) in axial (A) and coronal (B) planes demonstrating mild pericholecystic inflammatory changes of the gallbladder (arrows) without evidence of acute abnormality, biliary dilation, or perforation.

However, she re-presented 15 days later with worsening right upper quadrant pain and tenderness. A repeat CT scan showed mild inflammation, without any acute abnormality (Figure 3), raising suspicion of symptomatic cholecystitis. She was admitted for pain control.

**Figure 4:**
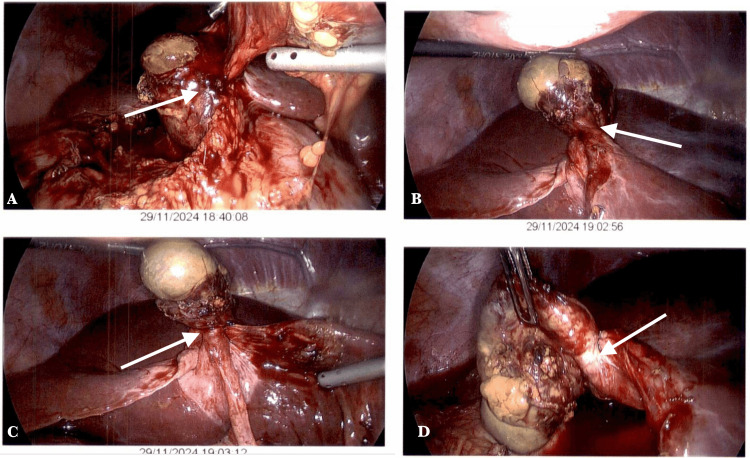
Laparoscopy images showing ischemic GB twisted at its pedicle and completely rotated clockwise at more than 180 degrees (A) Initial view showing a distended, dusky GB with a congested pedicle (arrow). (B) Rotation of the GB along its mesentery, with stretched and engorged cystic structures (arrow). (C) Further exposure revealing complete torsion with compromised vascular supply (arrow). (D) View of the GB after full identification of the twisted pedicle prior to detorsion (arrow). GB: Gallbladder

**Figure 5:**
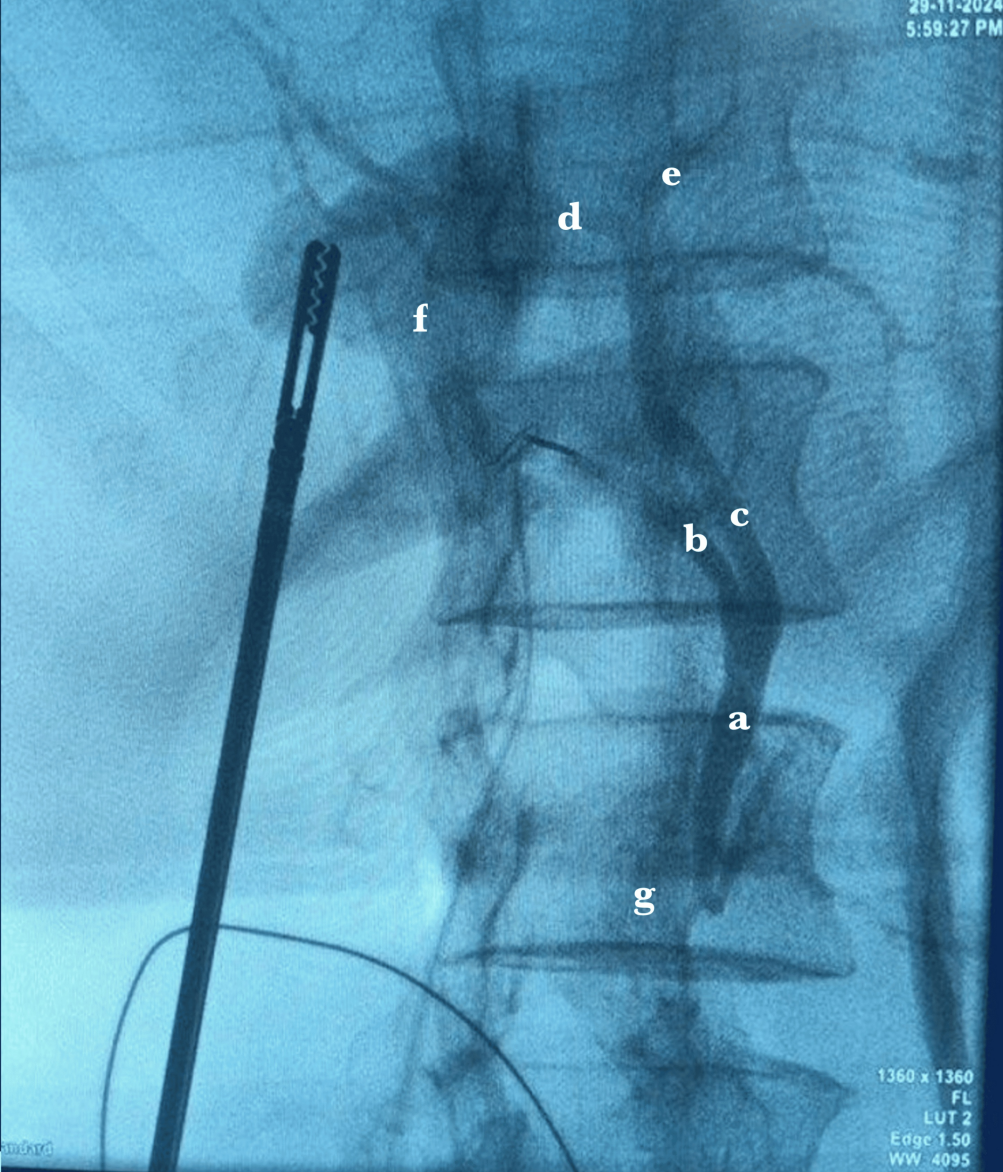
Intra-op cholangiogram showing non-opacification of the gallbladder in the presence of a patent biliary system Intraoperative cholangiogram demonstrating non-opacification of the gallbladder (f) despite a patent biliary system, including the common bile duct (a), cystic duct (b), common hepatic duct (c), right hepatic duct (d), and left hepatic duct (e). The duodenum (g).

The patient was classified as ASA II based on the American Society of Anesthesiologists (ASA) physical status classification. However, due to the lack of response to conservative management and worsening pain, emergency laparoscopy was scheduled. Intraoperatively, the gallbladder was found to be completely mobile and had undergone a 360° clockwise torsion (Figure 4). It appeared congested and ischemic. Following the achievement of the critical view of safety, an operative cholangiogram was conducted (Figure 5). The pedicle was identified, detorsed and removed laparoscopically without complications.

Gross examination of the excised gallbladder revealed eroded mucosa with fibrin. Following microscopy necrosis, bile extravasation, fibrosis, and hemorrhage were observed. The findings were consistent with ischemic injury secondary to gallbladder torsion. The patient had an uneventful postoperative recovery and was discharged in a stable condition the same day. At a three-month follow-up, she remained symptom-free; however, we acknowledge that this follow-up period is relatively short, and longer observation would be valuable to confirm sustained recovery and exclude late complications. Verbal informed consent was obtained from the patient for publication of this case report and properly documented; ethical approval was not required for a single-patient case study.

## Discussion

In the case presented, a 53-year-old woman was diagnosed intraoperatively with a 360° clockwise torsion of the gallbladder, representing a complete volvulus. Although gallbladder torsion predominantly affects elderly women aged 65 to 75 years, this patient’s presentation is not entirely atypical, being 53 years old. Similar rare cases have also been reported in children [10]. In the present case, known risk factors were present, most notably, a markedly low body mass index and unintentional weight loss of over six kilograms within the previous month prior to presentation. These findings are critical as visceral fat loss has been strongly implicated in increasing the risk of torsion, due to reduced cushioning and increased gallbladder mobility [9,11]. While our patient was younger than the average age group typically affected, the physiological state of visceral fat depletion and possible mesenteric laxity created an environment similar to that observed in older individuals.

Recent reports highlight the diagnostic challenge of gallbladder torsion, often mimicking acute cholecystitis until surgery confirms the diagnosis. Daly et al. (2023) described an elderly woman diagnosed intraoperatively after failed conservative treatment [12], while Cecire et al. (2021) reported two elderly thin women with acalculous cholecystitis initially misdiagnosed [13]. In contrast, Ren et al. (2024) identified torsion preoperatively in a pediatric case using magnetic resonance cholangiopancreatography (MRCP) [14]. Our case adds value by demonstrating torsion in a middle-aged patient with low BMI and improving radiological signs despite worsening symptoms, underscoring the need for clinical vigilance beyond the classic elderly profile (Table 1).

**Table 1:** Key features of selected recent cases of gallbladder torsion Summary of recent gallbladder torsion cases compared with the present case, highlighting key clinical features, imaging findings, and management outcomes. MRCP: Magnetic resonance cholangiopancreatography

Reference	Patient profile	Imaging/presentation	Management & outcome	Distinguishing feature vs current case
Daly et al., 2023 [12]	Woman in her 90s	Distended gallbladder, wall thickening, no stones	Laparoscopic cholecystectomy, uneventful	Very advanced age
Cecire et al., 2021 [13]	Two elderly thin women	Acalculous cholecystitis on imaging, torsion found intraoperatively	One percutaneous cholecystostomy failed → laparoscopic cholecystectomy	Initial attempt at non-operative intervention
Ren et al., 2024 (paediatric) [14]	Six-year-old girl	Floating gallbladder, MRCP identified torsion preoperatively	Laparoscopic cholecystectomy, good recovery	Pediatric age group and preoperative MRCP diagnosis
Current case	53-year-old woman, BMI 17.46, weight loss ~6.35 kg	CT: wall thickening, no stones or biliary dilation; improving radiology, worsening pain	CEPOD laparoscopic cholecystectomy, uneventful	Middle-aged, low BMI but not elderly; imaging improvement despite clinical worsening

Clinically, gallbladder torsion is difficult to distinguish from acute cholecystitis, particularly in its early stages. Most patients present with a sudden onset of right upper quadrant or epigastric pain, nausea, and vomiting. Fever and jaundice are often absent, and the patient may appear non-toxic. This constellation of symptoms is reflected in the “triad of triads” [4], which includes three groups of features: physical appearance (elderly, thin, spinal deformity), symptoms (sudden onset, upper abdominal pain, early vomiting), and examination findings (non-toxic presentation, palpable abdominal mass, pulse-temperature discrepancy). The index patient met five of the nine features (being thin, having a sudden onset of symptoms, early vomiting, right upper quadrant pain, and a non-toxic appearance), raising clinical suspicion despite the lack of classic radiologic signs.

Radiological imaging, while essential, often proves inconclusive in cases of gallbladder torsion. Ultrasound may show a distended or abnormally positioned gallbladder, and CT can reveal wall thickening, distension, or pericholecystic fluid, findings that closely mimic acute cholecystitis and can easily obscure the true diagnosis [4]. In our patient, the CT scan confirmed gallbladder distension and inflammation, but no gallstones or biliary dilatation were seen, leading to an initial interpretation of cholecystitis. The diagnosis of torsion was likely missed preoperatively because of the rarity of the condition and the subtlety of its imaging features.

On retrospective review, several clues might have suggested torsion: the absence of gallstones, the horizontal orientation of the gallbladder, and the clinical radiological mismatch, with improving imaging appearances despite worsening pain. Advanced modalities such as MRI or MRCP may occasionally identify the twisted cystic pedicle or whirl sign, but these are not always accessible or practical in emergency settings. Although diagnostic CT criteria including a floating gallbladder with surrounding fluid, horizontal alignment, and a twisted cystic duct have been described [7], such findings are inconsistently present. Consequently, as in our case, the definitive diagnosis is most often made intraoperatively.

Laboratory tests in gallbladder torsion are equally non-specific. Leukocytosis and mild elevations in liver enzymes, as seen in this case, are common in many inflammatory abdominal conditions. The absence of significant hyperbilirubinemia or obstructive features is typical, as the common bile duct is generally not involved in gallbladder torsion [11]. In the current case, the white blood cell count was suggestive of an inflammatory or ischemic process, possibly linked to early gangrenous changes while the elevated transaminases may reflect secondary hepatic stress due to localized inflammation.

However, the key turning point in this case was the patient’s failure to respond to conservative management despite worsening clinical symptoms and radiological improvement on re-presentation. In typical acute cholecystitis, symptoms usually improve within 24 to 48 hours of supportive therapy and antibiotics. Persistence or worsening of pain despite improving imaging findings should prompt consideration of complications such as gangrenous cholecystitis, empyema, or, as in this case, the rarer entity of gallbladder torsion. The decision to proceed with laparoscopy cholecystectomy was therefore both timely and pivotal in establishing the diagnosis and guiding definitive management.

Surgical exploration revealed a 360° clockwise rotation of the gallbladder on its axis, without any evidence of gallstones. The gallbladder appeared congested and ischemic but not perforated. This torsion accounted for the patient’s persistent pain and explained the failure of conservative management despite improving radiological signs of cholecystitis. Laparoscopic detorsion followed by cholecystectomy was successfully performed. This approach aligns with recommendations by Pottorf and colleagues [15], who advocated for laparoscopic management whenever feasible, as it reduces the risk of bile duct injury and allows for better visualization of surrounding structures. Histopathology in such cases usually reveals ischemic or gangrenous cholecystitis without the presence of gallstones. The absence of chronic inflammatory changes also helps differentiate it from long-standing calculous cholecystitis [8].

Postoperative recovery in our patient was uneventful, with rapid resolution of symptoms and normalization of inflammatory markers. The favorable outcome in this case underscores the importance of timely surgical intervention, before onset of gangrene or perforation. Delays in diagnosis and treatment can lead to gallbladder necrosis, perforation, bile peritonitis, and even death, with mortality rates reported at approximately 6% [3]. Therefore, clinicians should maintain a high index of suspicion for gallbladder torsion in patients presenting with features of acute cholecystitis but lacking the typical radiological or laboratory findings or those who fail to respond to standard treatment.

## Conclusions

Gallbladder torsion is a rare but critical cause of acute abdomen that often escapes early diagnosis. It should be considered in middle-aged and older female patients presenting with acute right upper quadrant pain, and low BMI, particularly when imaging is inconclusive and the clinical course is atypical. Laparoscopic exploration remains the gold standard for both diagnosis and treatment, with excellent outcomes when performed promptly. A high index of suspicion, early surgical intervention, and awareness of this condition can dramatically reduce morbidity and prevent potentially fatal complications.

## References

[1] Wendel AV (1898). VI. A case of floating gall-bladder and kidney complicated by cholelithiasis, with perforation of the gall-bladder. Ann Surg.

[2] Moser L, Joliat GR, Tabrizian P (2021). Gallbladder volvulus. Hepatobiliary Surg Nutr.

[3] Gupta V, Singh V, Sewkani A, Purohit D, Varshney R, Varshney S (2009). Torsion of gall bladder, a rare entity: a case report and review article. Cases J.

[4] Bouzas Cardaci M, Bivoleanu CV (2020). Gallbladder volvulus, a rare cause of acute abdomen, a case report. Int J Surg Case Rep.

[5] Shaikh AA, Charles A, Domingo S, Schaub G (2005). Gallbladder volvulus: report of two original cases and review of the literature. Am Surg.

[6] Ogut E, Yildirim FB, Memis O (2024). Duplicated gallbladder with acute cholecystitis: a case of unusual presentation and diagnostic challenges. World J Emerg Med.

[7] Layton B, Rudralingam V, Lamb R (2016). Gallbladder volvulus: it's a small whirl. BJR Case Rep.

[8] Kachi A, Nicolas G, Nasser J, Hashem M, Abou Sleiman C (2019). A rare presentation of gall bladder volvulus: a case report. Am J Case Rep.

[9] SK EW (1953). Torsion of the gall-bladder. Br Med J.

[10] Sun Y, Fang Z, Cao X (2024). Pediatric gallbladder torsion managed by laparoscopic cholecystectomy: a case report and scoping review. Front Pediatr.

[11] Gonzalez-Fisher RF, Vargas-Ramirez L, Rescala-Baca E, Dergal-Badue E (1993). Gallbladder volvulus. HPB Surg.

[12] Daly T, Byrne J, Aftab F (2023). Gallbladder torsion with gangrenous cholecystitis: a case report. J Surg Case Rep.

[13] Cecire J, Sutherland A, Das KK (2021). Gallbladder torsion masking as a calculus cholecystitis: a review of two cases including unsuccessful management with percutaneous cholecystostomy. J Med Cases.

[14] Ren H, Liu H, Liu X, Wei H, Tian P (2024). Case report: rare floating gallbladder torsion in a child. Front Med (Lausanne).

[15] Pottorf BJ, Alfaro L, Hollis HW (2013). A clinician’s guide to the diagnosis and management of gallbladder volvulus. Perm J.

